# CT-radiomics and clinical risk scores for response and overall survival prognostication in TACE HCC patients

**DOI:** 10.1038/s41598-023-27714-0

**Published:** 2023-01-11

**Authors:** Simon Bernatz, Oleg Elenberger, Jörg Ackermann, Lukas Lenga, Simon S. Martin, Jan-Erik Scholtz, Vitali Koch, Leon D. Grünewald, Yannis Herrmann, Maximilian N. Kinzler, Angelika Stehle, Ina Koch, Stefan Zeuzem, Katrin Bankov, Claudia Doering, Henning Reis, Nadine Flinner, Falko Schulze, Peter J. Wild, Renate Hammerstingl, Katrin Eichler, Tatjana Gruber-Rouh, Thomas J. Vogl, Daniel Pinto dos Santos, Scherwin Mahmoudi

**Affiliations:** 1 University Hospital Frankfurt, Department of Diagnostic and Interventional Radiology, Goethe University Frankfurt am Main, Theodor-Stern-Kai 7, 60590 Frankfurt am Main, Germany; 2 Dr. Senckenberg Institute for Pathology, University Hospital Frankfurt, Goethe University Frankfurt am Main, 60590 Frankfurt am Main, Germany; 3 Frankfurt Cancer Institute (FCI), 60590 Frankfurt am Main, Germany; 4 Department of Molecular Bioinformatics, Institute of Computer Science, Goethe University Frankfurt am Main, Robert-Mayer-Str. 11-15, 60325 Frankfurt am Main, Germany; 5 Department of Internal Medicine I, University Hospital Frankfurt, Goethe University, Frankfurt am Main, Germany; 6 Frankfurt Institute for Advanced Studies (FIAS), 60438 Frankfurt am Main, Germany; 7 Department of Diagnostic and Interventional Radiology, Faculty of Medicine and University Hospital Cologne, University of Cologne, Kerpener Str. 62, 50937 Cologne, Germany

**Keywords:** Cancer imaging, Hepatocellular carcinoma, Chemotherapy, Hepatocellular carcinoma, Biomarkers, Translational research

## Abstract

We aimed to identify hepatocellular carcinoma (HCC) patients who will respond to repetitive transarterial chemoembolization (TACE) to improve the treatment algorithm. Retrospectively, 61 patients (mean age, 65.3 years ± 10.0 [SD]; 49 men) with 94 HCC mRECIST target-lesions who had three consecutive TACE between 01/2012 and 01/2020 were included. Robust and non-redundant radiomics features were extracted from the 24 h post-embolization CT. Five different clinical TACE-scores were assessed. Seven different feature selection methods and machine learning models were used. Radiomics, clinical and combined models were built to predict response to TACE on a lesion-wise and patient-wise level as well as its impact on overall-survival prognostication. 29 target-lesions of 19 patients were evaluated in the test set. Response rates were 37.9% (11/29) on the lesion-level and 42.1% (8/19) on the patient-level. Radiomics top lesion-wise response prognostications was AUC 0.55–0.67. Clinical scores revealed top AUCs of 0.65–0.69. The best working model combined the radiomic feature LargeDependenceHighGrayLevelEmphasis and the clinical score mHAP_II_score_group with AUC = 0.70, accuracy = 0.72. We transferred this model on a patient-level to achieve AUC = 0.62, CI = 0.41–0.83. The two radiomics-clinical features revealed overall-survival prognostication of C-index = 0.67. In conclusion, a random forest model using the radiomic feature LargeDependenceHighGrayLevelEmphasis and the clinical mHAP-II-score-group seems promising for TACE response prognostication.

## Introduction

In 2020 primary liver cancer ranked as the third leading cause of cancer death world-wide^1^. Hepatocellular carcinoma (HCC) comprises around 75–85% of primary liver cancers and over the last 20 years its incidence has been rising^1,2^. The diagnostic work-up of HCC-suspicious observations includes among others clinical examinations, laboratory analysis, imaging studies and often tumor biopsy^2^. The treatment of HCC is complex and depends on the tumor stage. Potential curative treatments include liver resection, transplantation or local ablative methods like microwave ablation^2^. HCC is predominantly arterially vascularized enabling the intra-arterial application of chemotherapy and embolization^2^. These methods, like transarterial chemoembolization (TACE) are mainly palliative but may enable the complete destruction of the tumour or size-reduction to enable subsequent resection or transplantation (bridging therapy) in selected cases^2,3^. TACE can prolong patient’s overall survival (OS) but it may also harm patients with reduction of OS depending on patient selection^2^. A multitude of scores was developed to identify patients who will most likely benefit from TACE^2,4–8^. Nevertheless, the scores’ validity is scarce and the use for treatment decision making is not recommended outside clinical trials^2^. Consequently, patients are generally individually discussed in interdisciplinary tumor board meetings to define the appropriate therapy based on expert consensus. Recent emerges in the field of quantitative computational image analysis, termed radiomics, provide promising opportunities. Images are transformed in mineable data with subsequent bioinformatic analysis allowing lesion characterization beyond visual perception^9^. Radiomics’ prognostic and predictive potential was demonstrated in numerous cancer entities^9,10^. Only scarce evidence is available for TACE in HCC patients and most studies examined the pre-TACE contrast-enhanced MRI or CT though variant contrast agents or injection protocols might alter the results^11–14^. Lipiodol accumulation patterns after TACE might be used for response prognostication^15,16^ but to the best of our knowledge a high dimensional pattern quantification by means of radiomics was not performed yet.

We hypothesized that lipiodol retention patterns from the post-embolization CT after the first TACE can be quantified by means of radiomics to serve as imaging biomarkers for TACE response prediction. The aim of this study was to develop a predictive model for HCC patients on a (I) lesion-wise level, (II) patient-wise level and (III) for overall survival. Further, we aimed to stratify the best working model by comparing CT-derived features with clinical scores and a holistic combined model.

## Methods

Written informed consent was obtained from all patients and the study was approved by the institutional Review Boards of the University Cancer Center and the Ethical Committee at the University Hospital Frankfurt (project-number: SGI-10-2020). The patient population was not reported previously.

### Study design

**Figure 1:**
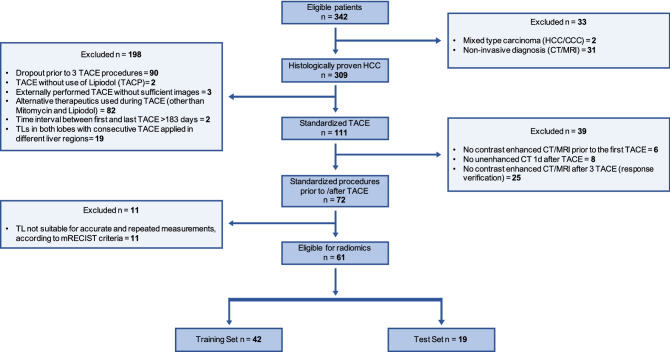
STARD Flowchart of patient inclusion into the study. STARD, Standards for Reporting Diagnostic Accuracy Studies.

In this retrospective study we consecutively enrolled 61 HCC-patients (female, 12; mean age, 65.3 ± 10.0 years) who were treated with conventional TACE between 01/2012 and 01/2020. Inclusion criteria were: (1) Histologically confirmed HCC, (2) three consecutive TACE exclusively with the therapeutics Mitomycin C (Medac®, Hamburg, Germany) and Lipiodol (Guerbet GmbH, France) ± degradable starch microspheres (EmboCept®S, PharmaCept GmbH, Berlin, Germany) and injected in the same liver region, (3) all mRECIST target lesions (TL) were treated with each TACE, (4) post-TACE unenhanced CT 24 h after TACE, (5) contrast-enhanced arterial and portal-venous/ delayed phase MRI or CT prior to the first and after the third TACE. Exclusion criteria: (1) Consecutive TACE applied in different liver regions, (2) time interval between first and last TACE > 6 months, (3) prior local therapy of TLs, (4) no TLs, (5) insufficient image quality, (6) other chemotherapeutic agents. 61 patients met the criteria and were evaluated. In Fig. 1 we depict the flow-chart of patient inclusion following STARD. A scheme of the study’s workflow is shown in Supplementary Data S1.

### Conventional TACE

Patients were treated with TACE in clinical routine as described in prior studies^17^ and in Supplementary Data S2. Imaging acquisition and examination parameters of the post-TACE CT are summarized in Supplementary Data S2.

### Assessment of tumor response

Response to TACE was assessed by mRECIST^18^. Lesion-wise response was defined as complete (CR) or partial response (PR) of TLs. Patient-wise response (CR or PR) was equivalent to the mRECIST overall response assessment^18^.

### Image segmentation and preprocessing

**Figure 2:**
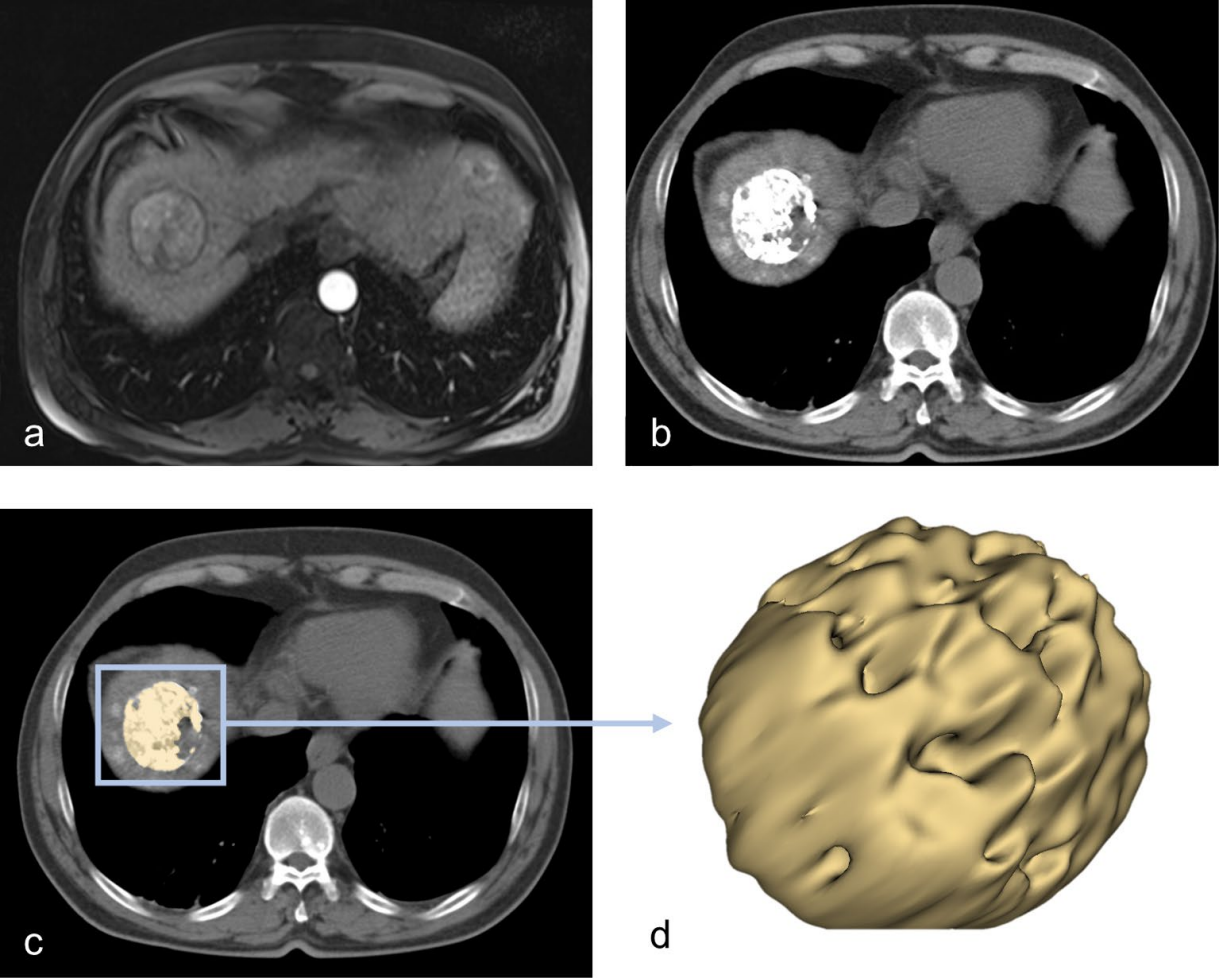
Workflow of the image analysis. (**a**) Baseline arterial-phase MRI showing mildly enhancing hepatocellular carcinoma. The 24 h post-TACE CT (**b**) was used to semi-automatically segment the lipiodol retention-pattern in three dimensions (**c**–**d**).

The image stack was visualized and processed using the 3D Slicer software platform (http://slicer.org, version 4.9.0)^19,20^. We resampled the images to a spacing of 1 mm × 1 mm × 1 mm prior to features extraction. One blinded investigator (OE, board-certified radiologist, 10 years of experience) tagged and segmented a maximum of two TLs per patient using the 24 h post-embolization CT after the first TACE. The tagged TLs were independently segmented by a second blinded investigator (SB, radiologist-in-training, 3.5 years of experience). Segmentation was performed as follows: a three-dimensional volume of interest (VOI) was manually drawn in the HCC-lesion, sparing equivocal border zones. The semi-automatic grow from seeds algorithm was used to augment the VOI to match the whole tumor habitat^20–22^. Clear foci of segmentation error were manually erased using the brush-erase tool. A representative segmentation is shown in Fig. 2.

### Radiomic analysis

We used PyRadiomics within 3D Slicer for radiomics features extraction^20,23^. With default settings, all original standard features were extracted (n = 107) as described in prior studies^24^. The radiomics quality score was 14 (https://radiomics.world/rqs, Supplementary Data S3)^25^.

### Inter-observer robustness and feature redundancy

The intra-class correlation coefficient (ICC) was calculated for each feature using ICC3 of the Pingouin package^24,26^. ICC values were interpreted with thresholds commonly used in ICC-analysis, i.e. ICC 0.75–1 = excellent^24^. We discarded all features with ICC < 0.75 (n = 8) (Supplementary Data S4 and S5). We inter-correlated the robust features by Pearson method and excluded all highly correlated (Pearson > 0.95) features (n = 52) (Supplementary Data S6).

### Clinical benchmark

We calculated five different clinical scores for the assessment of the liver function in HCC and for TACE response prediction as described in Supplementary Data S7. The degree of TL’s hypervascularization was visually assessed by three independent raters (see Supplementary Data S7).

### Imaging biomarker selection and model development

We describe the workflow of feature selection and model development in a scheme in Supplementary Data S1 and in detail in Supplementary Data S8. We performed all analysis in Python 3.7.6. We used StandardScaler^27^ to scale the data to uniform variance. We used t-distributed stochastic neighbor embedding (t-SNE) plots to explore cluster distributions (scikit-learn^27^). We split our dataset into 70% training and 30% testing on a patient level using GroupShuffleSplit^27^. Fist, we assessed the lesion-wise response using seven different feature selection strategies and seven different machine learning models with hyperparameter optimization using Hyperopt^28^ (see supplementary Data S8). Feature selection and model development was individually done for radiomics features, clinical scores and their combination. This approach ensured that the radiomics model was benchmark against clinical and combined models. The best working model was locked and transferred to predict the response on the patient-level. The selected features were used to train a random survival forest for overall survival prediction using Scikit-survival 0.16.1^29^. The performance was assessed by the concordance-index. We used the lifelines package ^30^ to build and compare Kaplan Meier curves. The WORC.statistics package^31^ was used for the DeLong’s test. For graphical illustrations Python 3.7.6. and Affinity Designer (Serif (Europe) Ltd) was used.

### Ethical approval

Patient data used in this study was provided by the University Cancer Center Frankfurt (UCT). Written informed consent was obtained from all patients and the study was approved by the institutional Review Boards of the UCT and the Ethical Committee at the University Hospital Frankfurt (project-number: SGI-10–2020). All analysis were performed in accordance with relevant guidelines and regulations.

## Results

### Study population

Our dataset comprised 61 patients (mean age, 65.3 years ± 10.0 [SD]; 12 women) with 94 HCC mRECIST TLs. 38.3% (36/94) of the TLs and 41.0% (25/61) of the patients had response to TACE. We randomly drew 70% of the patients (n = 42, mean age, 66.1 years ± 10.3 [SD]) with 65 TLs as training and 30% of the patients (n = 19, mean age, 63.5 years ± 9.2 [SD]) with 29 TLs as independent testing set. Response to therapy was seen in 40.5% (17/42) training-patients (38.5% (25/65) training-TLs) and in 42.1% (8/19) testing-patients (37.9% (11/29) testing-TLs). We depict the overall survival for the complete cohort, training and testing sets in Supplementary Data S9. Patient demographic characteristics are shown in Table 1. Flow diagram of patient inclusion is shown in Fig. 1.

**Table 1:** Clinical and epidemiological characteristics.

Demographic variables	All	Train	Test	*p*-value
Patients (n)	61	42	19	
Sex, male (%)	49 (80.3)	35 (83.3)	14 (73.7)	0.380
Median age at diagnosis (years)	66 (37–86)	67 (37–86)	63 (50–86)	0.323
Median time diagnosis to TACE (days)	43 (3–978)	42 (3–874)	59 (17–978)	0.252
Median size of Target lesions (cm)	2.9 (1.0–10.1)	2.2 (1.0–9.8)	3.4 (1.0–10.1)	0.143
Cause of HCC (%)				0.968
Hepatitis B	9 (14.8)	6 (14.3)	3 (15.8)	
Hepatitis C	14 (23.0)	10 (23.8)	4 (21.1)	
Alcohol	19 (31.1)	14 (33.3)	5 (26.3)	
NASH	5 (8.2)	3 (7.1)	2 (10.5)	
Alcohol + viral Hepatitis	6 (9.8)	4 (9.5)	2 (10.5)	
Alcohol + NASH	1 (1.6)	1 (2.4)	0 (0)	
Others (cryptogenic cirrhosis, AIH)	7 (11.5)	4 (9.5)	3 (15.8)	
BCLC prior to TACE				0.092
A	18 (29.5)	14 (33.3)	4 (21.1)	
B	33 (54.1)	24 (57.1)	9 (47.4)	
C	10 (16.4)	4 (9.5)	6 (31.6)	
D	0 (0.0)	0 (0.0)	0 (0.0)	
Child Pugh Score prior to TACE				0.644
A	36 (59.0)	25 (59.5)	11 (57.9)	
B	6 (9.8)	5 (11.9)	1 (5.3)	
C	0 (0.0)	0 (0.0)	0 (0.0)	
N/A	19 (31.1)	12 (28.6)	7 (36.8)	
MELD-Score prior to TACE				0.141
< 6	1 (1.6)	0 (0.0)	1 (5.3)	
< 10	23 (37.7)	13 (31.0)	10 (52.6)	
< 15	11 (18.0)	10 (23.8)	1 (5.3)	
< 20	1 (1.6)	1 (2.4)	0 (0.0)	
N/A	25 (41.0)	18 (42.9)	7 (36.8)	
Median albumin (g/dl)	3.8 (1.8–7.2)	3.8 (1.8–7.2)	3.9 (3.0–4.5)	0.686
Median bilirubin (mg/dl)	0.8 (0.3–2.2)	1.1 (0.3–2.2)	0.9 (0.3–1.3)	0.540
Median INR	1.1 (0.9–3.3)	1.1 (0.9–3.3)	1.1 (1.0–1.8)	0.204
Median CRP (mg/dl)	0.4 (0.03–4.4)	0.5(0.03- 4.4)	0.3 (0.1–2.9)	0.632
Median AFP (ng/ml)	12.9 (2.1–60,500.0)	9.2 (2.1–60,500.0)	17.3 (2.2–9276.0)	0.615

The train and test set were statistically analyzed using the Pearson Chi-Square test or two-sided t-test for ordinal or continuous outcomes.

### Interobserver robustness and feature redundancy

The mean intra-class correlation coefficient was 0.90 for all feature classes combined, ranging from 0.76 (± 0.41, ngtdm) to 0.98 (± 0.03, firstorder) (Supplementary Data S4). A set of 8 features (marked in bold in Supplementary Data S5) revealed ICC values < 0.75 (range: 0.04–0.74) and were excluded for further analysis. We intercorrelated the remaining robust features with Pearson metric to exclude 52 features due to redundancy. The final robust and non-redundant feature set consisted of 47 features (Supplementary Data S6).

### Lesion-wise response characterization using dimensionality reduction

To assess the variance of radiomics and clinical features regarding the individual TL response to TACE, we used low dimensional embedding via t-SNE plots for each feature subset (radiomics, clinical features and their combination). Neither feature subset showed clear clusters of response (Supplementary Data S10). Therefore, we pursued our analysis with models of higher complexity.

### Lesion-wise response prognostication: feature selection, model development and clinical benchmarking

**Figure 3:**
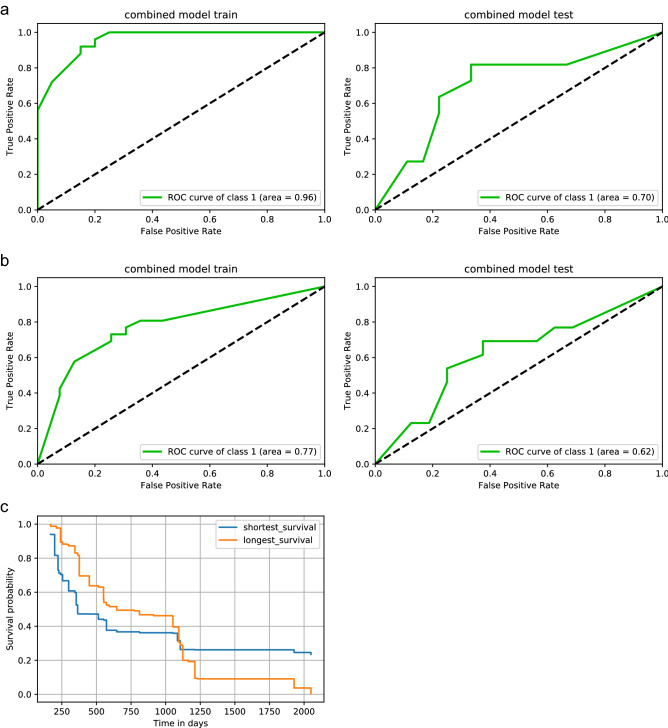
Prediction of response and overall survival. (**a**, **b**) Receiver operating characteristics (ROC) curves trained and tested using the final combined feature set of the radiomics feature LargeDependenceHighGrayLevelEmphasis and the clinical score mHAP_II_score_group. (**a**) Lesion-wise prediction with class 1 describing the individual responding lesions according to mRECIST. (**b**) Patient-wise prediction with class 1 describing the overall response on the patient-level according to mRECIST, including the impact of non-target lesions and potential new-lesions. (**c**) Patient-wise overall survival prediction. Kaplan–Meier plot of two test-patients who showed the shortest (102 days) or longest (censored at 2043 days) survival. Kaplan–Meier estimator was based on our final model. Logrank-Test was used.

The feature selection and model training were applied independently on three different feature subsets: (I) radiomics features, (II) clinical features or (III) their combination. We identified prognostic signatures for each subset (Table 2, Supplementary Data S8). For each subset, we validated the model on our hold-out test set to stratify the best working model using ROC AUC metrics. If models showed equal performance, we ranked models higher the less features they needed for the prediction. The best working radiomics model revealed a test AUC of 0.60 (train AUC = 1.00). The best clinical model reflected bias with a better test than train performance (train/ test AUC = 0.61/ 0.69). The combined clinical and radiomics model showed the best performance with test AUCs of 0.70 (train AUC = 0.96) (Fig. 3a, Table 3). This best performing combined model was a Random Forest Classifier which included the CT-derived radiomics feature LargeDependenceHighGrayLevelEmphasis and the clinical score mHAP_II_score_group. This final prognostic model was locked (Supplementary Data S8).

**Table 2:** Feature subsets of different selection strategies.

Feature selection	Selected features
	Radiomics
RFE	Flatness, Sphericity, 10Percentile, Maximum, Skewness, Imc1, LargeDependenceHighGrayLevelEmphasis, LargeAreaEmphasis, LargeAreaLowGrayLevelEmphasis
RFA	LargeDependenceHighGrayLevelEmphasis
LASSO	Flatness, Minimum, Skewness, LargeAreaLowGrayLevelEmphasis
	clinical scores
RFE & RFA	mHAP_II_score_group
LASSO	mHAP_II_score_group, 6_and_12_group
	Combined features (clinical and radiomics)
Best combined	LargeDependenceHighGrayLevelEmphasis, mHAP_II_score_group

LASSO, least absolute shrinkage and selection operator; RFA, recursive feature addition; RFE, recursive feature elimination. See Supplementary Data S8 for more information.

**Table 3:** Classifier, feature selection strategy and performance of the best lesion-wise models.

Classifier	Selection	Train		Test	
		Accuracy	AUC	Accuracy	AUC
Radiomics					
GradientBoostingClassifier	RFE	1.000	1.000	0.517	0.596
ExtraTreesClassifier	RFA	0.738	0.881	0.586	0.674
AdaBoostClassifier	LASSO	1.000	1.000	0.586	0.551
Clinical scores					
SVC	RFE & RFA	0.615	0.606	0.621	0.689
SGDClassifier	LASSO	0.615	0.618	0.621	0.649
Combined features					
RandomForestClassifier	Best combined	0.862	0.957	0.724	0.705

AUC, area under the curve; LASSO, least absolute shrinkage and selection operator; RFA, recursive feature addition; RFE, recursive feature elimination. See Supplementary Data S8 for more information.

### Patient-wise response prognostication: model transferability and prognostication of overall survival

We transferred our locked lesion-wise model on a patient-wise level. Response to TACE was defined according to mRECIST including the effect of non-target or potentially new lesions. The model demonstrated a prognostic performance of AUC test = 0.62, CI = 0.41–0.83 (AUC train = 0.77, CI = 0.68–0.91) (Fig. 3b). We transferred the combined CT-derived and clinical two-feature set to test the prognostication of overall survival. The model yielded a C-index of 0.67 (C-index train = 0.71) for overall survival prognostication compared to a C-index of 0.58 (train: 0.70) or 0.60 (train: 0.60) using only the single clinical or single radiomic feature. Finally, we selected two test-patients who showed the shortest (102 days) or longest (last living contact at 2043 days) survival to estimate their individual survival using the Kaplan–Meier estimator based on our final model. We computed the risk score that represents the expected number of events for a particular terminal node in the forest for the respective test patients. The patient with short survival yielded a higher risk score (26.89) than the patient with long overall survival (23.55). We depict the predicted Kaplan–Meier plot of the two patients in Fig. 3c which revealed significant difference in the logrank-Test (*p* = 0.006).

## Discussion

In this study, we assessed the utility of machine learning models in predicting response to repetitive TACE in HCC patients. We used Lipiodol-retention radiomics of the first post-TACE control CT as imaging biomarker. We applied multiple feature selection strategies to train a multitude of machine learning models with exhaustive hyperparameter optimization to stratify tumor lesions’ response to TACE. We transferred our lesion-wise model to a patient-level and corroborated our findings by overall survival prognostication. We demonstrated the model’s ability to denote tumor risk scores associated with shorter or longer overall survival. CT-derived features were benchmarked against clinical risk scores and the best working model consisted of the combination of the single radiomics feature LargeDependenceHighGrayLevelEmphasis and the single clinical risk score mHAP_II_score_group.

HCC hallmark imaging characteristics (arterial hyperenhancement with portal venous/ delayed wash-out) and mRECIST assessment of viable tumour components are well established, especially in patients treated with TACE^2^. Recent studies aimed to stratify imaging biomarkers extracted from pre-treatment contrast-enhanced imaging to build predictive models for HCC TACE response^11–14^. The studies tended to build holistic nomograms including imaging and clinical features and yielded promising predictive performances of overall survival ranging from C-indices of 0.70 to 0.77 which are in a similar range to our results^11,13,14^. Kuang et al. yielded lesion-wise mRECIST response predictions of AUC approx. 0.81 using pre-treatment MRI and clinical data^12^. No patient-wise or survival analysis was done and it remained unclear how many TACE were applied prior to the analysis^12^. We followed a more stringent approach by building a model starting at a lesion-wise prediction, transferring the model to a patient-wise level and finally to overall survival. Further, arterial-phase imaging might suffer from reduced image quality due to artifacts or poor arterial phase capture. This might limit the development of robust AI models as they add noise to a system which already suffers from robustness deficiencies even in an experimental setting^24,32,33^. In line with prior studies ^15,16^, our results promote the potential of lipiodol deposits to serve as imaging biomarker. Miszczuk et al. ^16^ prospectively enrolled 39 liver cancer patients (n = 22, HCC) treated with TACE and they could show, that high Lipiodol coverage on the 24-h post-TACE CT was associated with response to therapy. Lipiodol retention may serve as a surrogate for arterial hyperenhancement ^16^, the vascularization pattern of HCC lesions might have prognostic impact ^34^ and our results provide quantitative corroboration of these findings. In our model, the GLDM feature LargeDependenceHighGrayLevelEmphasis, which depends on higher gray-level values (https://pyradiomics.readthedocs.io/), had the highest predictive impact. This is in line with Brancato et al.^35^ who predicted histological HCC grade by means of radiomics. The feature LargeDependenceHighGrayLevelEmphasis was contributing to the most powerful model to differentiate histological grade 1 versus grade 3 tumors^35^ emphasizing the feature’s potential to serve as imaging biomarker for HCC aggressiveness. The current ESMO clinical practice guidelines for hepatocellular carcinoma^2^ do not recommend the use of prognostic scores for treatment algorithms outside clinical trials and they describe only the hepatoma arterial-embolisation prognostic (HAP) score as potential stratification tool for TACE in the future^2^. This is in line with the results of our study as the best performing clinical scores revealed biased train-/test results. Nevertheless, the holistic model combining LargeDependenceHighGrayLevelEmphasis with the modified HAP-II^6^ score improved the models’ performances and established the best working model. Our study has limitations that warrant discussion. The retrospective nature of our study might impose selection bias. With 61 patients and 94 lesions our study population is rather small which might lower generalizability, but our cohort is very homogenous only including patients with histologically confirmed HCC, a total of three TACE prior to response assessment and usage of the same chemotherapeutic agent in each patient. In approx. 20% of patients additional degradable starch microspheres (EmboCept®S, PharmaCept GmbH, Berlin, Germany) were given which might have altered the retention in our standard-of-care real-world population. We leveraged a multitude of feature selection and classification strategies, nevertheless various degrees of overfitting were present in some models. Though we resampled the images to a spacing of 1 × 1 × 1 mm, we used standard-of-care imaging to develop our models with post-embolization CTs with originally 5 mm slice thickness and availability of true 1 mm reconstructions would have been favorable.

In conclusion, radiographic features derived from standard-of-care 24 h post-embolization CT have the potential to serve as imaging biomarkers for prognostication of response to TACE in HCC patients. Imaging biomarkers and clinical risk scores seem to incorporate complementary prognostic information and a combined final model of a clinical risk score and a single radiomics feature revealed the best performance. This emerging approach might pave the way to aiding clinical decision making in a clinical domain currently dominated by subjective expert consensus. Such tools might enable the more accurate stratification of patients for personalized healthcare avoiding potential adverse events in patients who most likely won’t respond to TACE.

## Supplementary Information

Supplementary Information.

## Data Availability

The datasets generated and/or analysed during the current study are not publicly available due to privacy regulations but are available from the corresponding author on reasonable request.

## Abbreviations

CR: Complete response
DART: Dropouts meet multiple additive regression trees
DICOM: Digital Imaging and Communications in Medicine
GBDT: Gradient boosting decision tree
GLCM: Gray level co-occurrence matrix
GLRLM: Gray level run length matrix
GLSZM: Gray level size zone matrix
GLDM: Gray level dependence matrix
GOSS: Gradient-based one-side sampling
HCC: Hepatocellular carcinoma
ICC: Intra-class correlation coefficient
LASSO: Least absolute shrinkage and selection operator
mRECIST: Modified Response Evaluation Criteria in Solid Tumors
NGTDM: Neighboring gray tone difference matrix
NTL: Non-target lesion
PD: Progressive disease
PR: Partial response
OS: Overall survival
RF: Random forest
RFA: Recursive feature addition
RFE: Recursive feature elimination
SD: Stable disease
SGD: Stochastic gradient descent
STARD: Standards for Reporting Diagnostic Accuracy Studies
SVC: Support vector classifier
TACE: Transarterial chemoembolization
TL: Target lesion
T-SNE: T-distributed stochastic neighbor embedding
VOI: Volume of interest
