# Supplementary material for: CT-radiomics and clinical risk scores for response and overall survival prognostication in TACE HCC patients
Source: Sci Rep. 2023 Jan 11;13:533. doi: 10.1038/s41598-023-27714-0 (PMC9834236; doi:10.1038/s41598-023-27714-0)
Supplement: Supplementary file 1 — Supplementary Information. [file 41598_2023_27714_MOESM1_ESM.docx]

**CT-radiomics and clinical risk scores for response and overall survival prognostication in TACE HCC patients.**

**Supplementary Material**

Simon Bernatz^1^^,2,3,#^, MD; Oleg Elenberger^1^, Mr; Jörg Ackermann^4^, PhD; Lukas Lenga^1^, MD; Simon S. Martin^1^, MD; Jan-Erik Scholtz^1^, MD; Vitali Koch^1^, MD; Leon D. Grünewald^1^, MD; Yannis Herrmann^1^, Mr; Maximilian N. Kinzler^5^, MD; Angelika Stehle^5^, MD; Ina Koch^4^, PhD; Stefan Zeuzem^5^, MD; Katrin Bankov^2^, PhD; Claudia Doering^2^, PhD; Henning Reis^2^, MD; Nadine Flinner^2^, PhD; Falko Schulze^2^, MD; Peter J. Wild^2,3,6^, MD; Renate Hammerstingl^1^, MD; Katrin Eichler^1^, MD; Tatjana Gruber-Rouh^1^, MD; Thomas J. Vogl^1^, MD; Daniel Pinto dos Santos^1,7^, MD; Scherwin Mahmoudi^1^, MD

^1^Goehte University Frankfurt am Main, University Hospital Frankfurt, Department of Diagnostic and Interventional Radiology, Theodor-Stern-Kai 7, 60590 Frankfurt am Main, Germany;

^2^Dr. Senckenberg Institute for Pathology, University Hospital Frankfurt, Goethe University Frankfurt am Main, 60590, Frankfurt am Main, Germany;

^3^Frankfurt Cancer Institute (FCI), 60590, Frankfurt am Main, Germany;

^4^Department of Molecular Bioinformatics, Institute of Computer Science, Johann Wolfgang Goethe-University, Robert-Mayer-Str. 11-15, 60325 Frankfurt am Main, Germany;

^5^Department of Internal Medicine I, University Hospital Frankfurt, Goethe University Frankfurt am Main, Germany;

^6^Frankfurt Institute for Advanced Studies (FIAS), 60438, Frankfurt am Main, Germany;

^7^Department of Diagnostic and Interventional Radiology, University of Cologne, Faculty of Medicine and University Hospital Cologne, Kerpener Str. 62, 50937, Cologne, Germany.

# Corresponding author.

[**S1. Workflow of model development** 4](#_Toc116972045)

[**S2. Conventional TACE** 5](#_Toc116972046)

[**S3. Radiomics quality score** 7](#_Toc116972047)

[**S4. Intraclass correlation analysis: radiomics feature classes** 10](#_Toc116972048)

[**S5. Intraclass correlation analysis: individual radiomics features** 11](#_Toc116972049)

[**S6. Pearson correlation analysis** 14](#_Toc116972050)

[**S8. Feature selection and model development** 19](#_Toc116972051)

[**S9. Overall survival** 21](#_Toc116972052)

[**S10. Low dimensional embedding did not identify response clusters** 22](#_Toc116972053)

[**References** 23](#_Toc116972054)

# **S1. Workflow of model development**


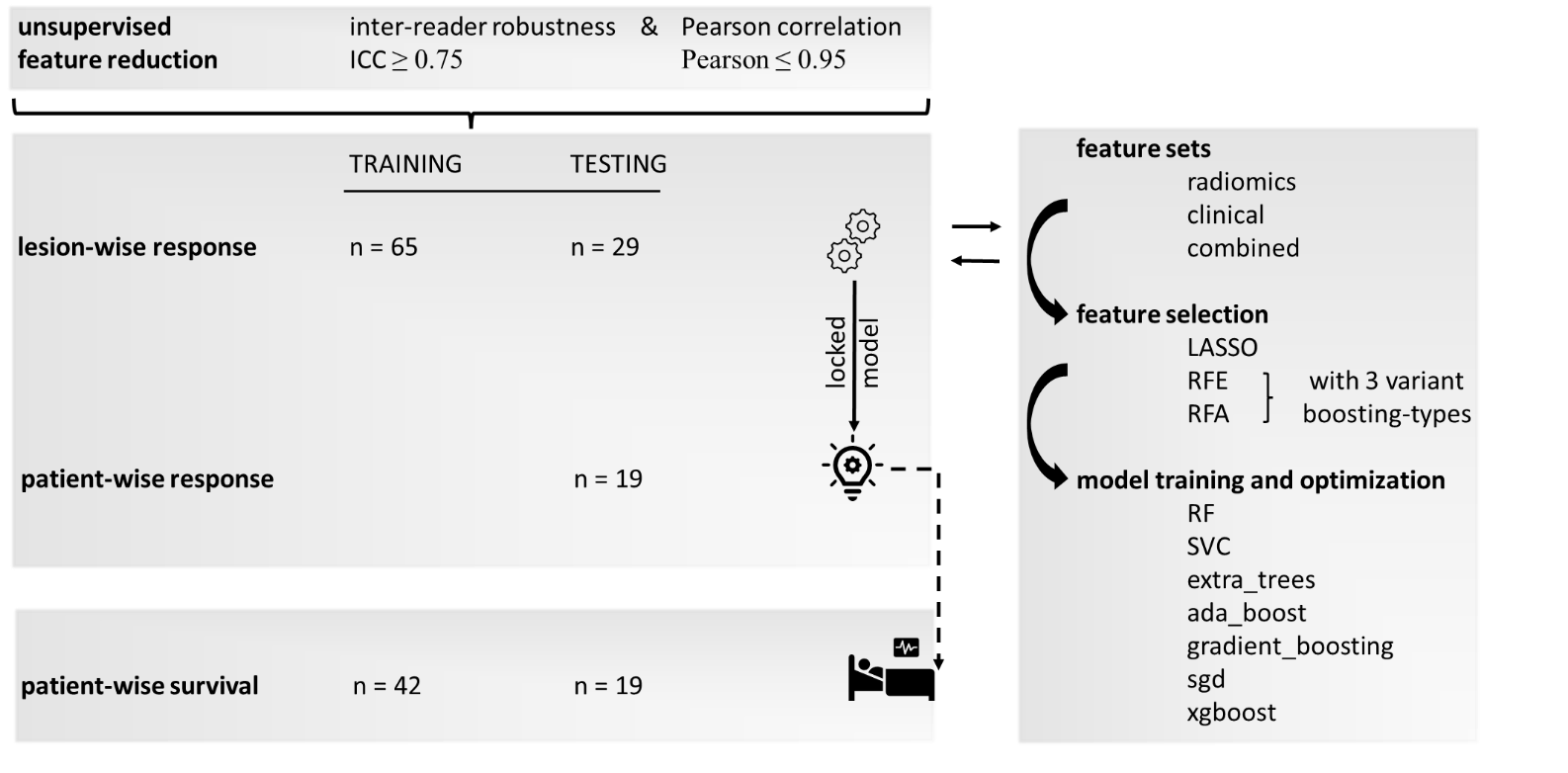


**Supplementary Figure 1.** Workflow of model development

ICC, intra-class correlation coefficient; LASSO, least absolute shrinkage and selection operator; RF, random forest; RFA, recursive feature addition; RFE, recursive feature elimination; SGD, stochastic gradient descent classifier; SVC, C-support vector classifier.

# **S2. Conventional TACE**

Following standard procedures, the tumor-feeding vasculature was visualized, and a catheter was positioned in the most distal achievable tumor-feeding segmental or subsegmental artery in super-selective technique to inject the therapeutics. If both liver lobes were affected, the lobe with higher tumor burden was treated as determined by baseline MRI. For the cTACE Mitomycin C (Medac®, Hamburg, Germany) was carefully administered (maximum of 8 mg/ m^2^ body surface (mean: 9.87 mg ± 0.52)) as a mixture with the embolization agent Lipiodol (Guerbet GmbH, France). In 19.7% (36/183) of TACE-procedures (29.5% (18/61) of patients) additional degradable starch microspheres (EmboCept®S, PharmaCept GmbH, Berlin, Germany) (mean: 182.63mg ± 84.97) were given in at least one TACE. Lipiodol retention was evaluated in an unenhanced CT performed one day after every cTACE. The unenhanced CT acquisition protocols operated the x-ray tubes at 120 ± 13.5 kilovoltage and 213 ± 54.7 mean mAs. We obtained a mean volume CT dose index of 10.2 ± 6.5 mGy and a mean dose-length product of 238.4 ± 153.1 mGy x cm. The images were reconstructed in clinical routine with a slice thickness of 5 mm in axial plane. Three repetitive cTACE were performed in each patient prior to response assessment in a maximum timeframe of 6 months. In the following figure the baseline MRI, TACE procedure, 24h post-TACE CT and post 3 TACE follow-up MRI of a representative patient is shown.


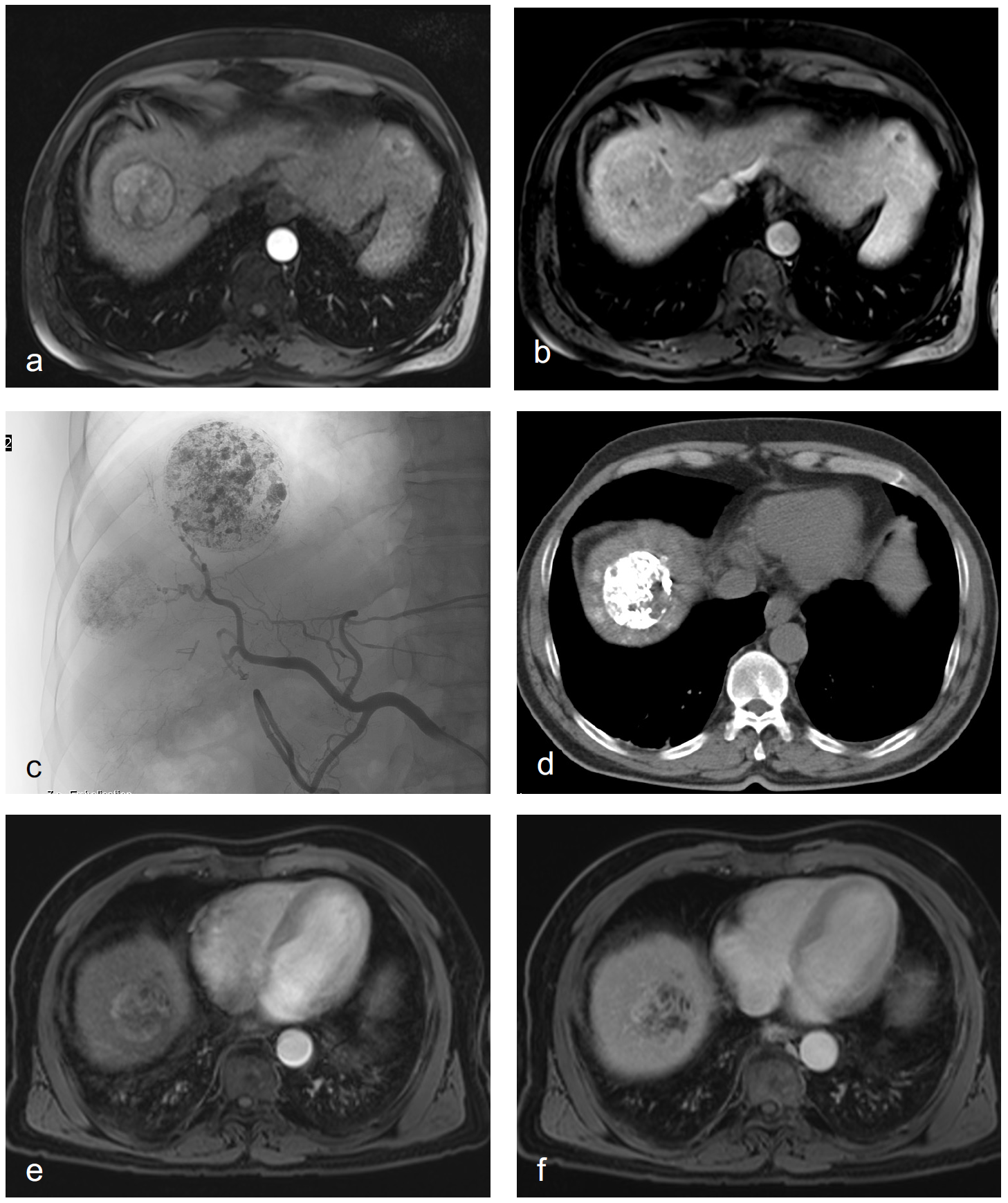


**Supplementary Figure 2.** Conventional TACE with baseline and follow-up imaging

Baseline MRI in arterial (a) and portal-venous (b) phase. Representative images of the first cTACE procedure (c) and respective 24h post-embolization CT (d). Follow-up MRI after the third cTACE in arterial (e) and portal-venous (f) phase.

# **S3. Radiomics quality score**

**Supplementary Figure 3.** Radiomics quality score of the study

Assessment of the radiomics quality score (<https://radiomics.world/rqs>). ^1^

# **S4. Intraclass correlation analysis: radiomics feature classes**

**Supplementary Figure 4.** Intraclass correlation analysis of the radiomics feature classes

Box-Whisker Plots depicting the intraclass correlation analysis for each radiomic feature class.

| ICC3 | mean | std |
| --- | --- | --- |
| firstorder | 0.978 | 0.027 |
| glcm | 0.958 | 0.051 |
| gldm | 0.898 | 0.127 |
| glrlm | 0.897 | 0.129 |
| glszm | 0.905 | 0.093 |
| ngtdm | 0.758 | 0.413 |
| shape | 0.925 | 0.086 |

**Supplementary Table 1.** Intraclass correlation coefficient for each radiomic feature class

For each radiomic feature class the mean ± standard deviation intraclass correlation coefficient is depicted.

# **S5. Intraclass correlation analysis: individual radiomics features**

**Supplementary Figure 5.** Intraclass correlation analysis for each radiomics feature

Swarmplot depicting the intraclass correlation analysis for each radiomics feature

| **class** | **features** | **ICC3** |
| --- | --- | --- |
| firstorder | Maximum | 0.997 |
| firstorder | 90Percentile | 0.995 |
| glcm | DifferenceAverage | 0.995 |
| glcm | Contrast | 0.995 |
| glcm | SumSquares | 0.995 |
| firstorder | MeanAbsoluteDeviation | 0.994 |
| glcm | ClusterTendency | 0.994 |
| firstorder | Variance | 0.994 |
| gldm | GrayLevelVariance | 0.994 |
| glszm | SizeZoneNonUniformity | 0.994 |
| glszm | GrayLevelVariance.2 | 0.994 |
| glrlm | GrayLevelVariance.1 | 0.993 |
| firstorder | Energy | 0.993 |
| firstorder | TotalEnergy | 0.993 |
| glszm | GrayLevelNonUniformity.2 | 0.993 |
| firstorder | Range | 0.993 |
| firstorder | RobustMeanAbsoluteDeviation | 0.993 |
| glcm | DifferenceEntropy | 0.992 |
| firstorder | RootMeanSquared | 0.991 |
| glcm | DifferenceVariance | 0.991 |
| firstorder | InterquartileRange | 0.991 |
| glcm | Id | 0.991 |
| gldm | SmallDependenceEmphasis | 0.991 |
| glcm | Idm | 0.991 |
| glcm | JointEntropy | 0.990 |
| ngtdm | Contrast.1 | 0.989 |
| glszm | ZonePercentage | 0.989 |
| ngtdm | Complexity | 0.989 |
| gldm | DependenceNonUniformityNormalized | 0.988 |
| firstorder | Entropy | 0.988 |
| ngtdm | Strength | 0.988 |
| glcm | SumEntropy | 0.987 |
| glrlm | RunLengthNonUniformity | 0.987 |
| firstorder | Mean | 0.987 |
| glcm | ClusterShade | 0.987 |
| glcm | InverseVariance | 0.986 |
| glcm | ClusterProminence | 0.985 |
| glrlm | RunLengthNonUniformityNormalized | 0.985 |
| glrlm | ShortRunEmphasis | 0.983 |
| gldm | DependenceNonUniformity | 0.983 |
| shape | LeastAxisLength | 0.983 |
| firstorder | Median | 0.982 |
| glrlm | RunPercentage | 0.981 |
| firstorder | Uniformity | 0.978 |
| gldm | LargeDependenceEmphasis | 0.975 |
| glrlm | GrayLevelNonUniformityNormalized | 0.975 |
| shape | VoxelVolume | 0.974 |
| shape | MeshVolume | 0.974 |
| glrlm | RunEntropy | 0.974 |
| glcm | MaximumProbability | 0.973 |
| shape | MinorAxisLength | 0.972 |
| shape | SurfaceArea | 0.971 |
| gldm | SmallDependenceHighGrayLevelEmphasis | 0.970 |
| gldm | DependenceEntropy | 0.969 |
| shape | Maximum2DDiameterSlice | 0.967 |
| glcm | JointEnergy | 0.966 |
| gldm | DependenceVariance | 0.964 |
| glrlm | LongRunEmphasis | 0.961 |
| glrlm | GrayLevelNonUniformity.1 | 0.959 |
| firstorder | Kurtosis | 0.957 |
| shape | MajorAxisLength | 0.955 |
| glrlm | RunVariance | 0.953 |
| glcm | Imc2 | 0.953 |
| glszm | LargeAreaHighGrayLevelEmphasis | 0.952 |
| firstorder | Skewness | 0.952 |
| shape | Maximum3DDiameter | 0.951 |
| shape | Maximum2DDiameterRow | 0.951 |
| gldm | GrayLevelNonUniformity | 0.950 |
| shape | Maximum2DDiameterColumn | 0.948 |
| glszm | SizeZoneNonUniformityNormalized | 0.946 |
| glszm | SmallAreaEmphasis | 0.946 |
| glszm | ZoneEntropy | 0.945 |
| glszm | SmallAreaHighGrayLevelEmphasis | 0.945 |
| glcm | Correlation | 0.943 |
| glszm | GrayLevelNonUniformityNormalized.1 | 0.937 |
| firstorder | 10Percentile | 0.936 |
| glszm | HighGrayLevelZoneEmphasis | 0.934 |
| glcm | MCC | 0.930 |
| glcm | Idn | 0.927 |
| shape | SurfaceVolumeRatio | 0.924 |
| glcm | JointAverage | 0.923 |
| glcm | SumAverage | 0.923 |
| glcm | Imc1 | 0.922 |
| glrlm | ShortRunHighGrayLevelEmphasis | 0.906 |
| shape | Sphericity | 0.906 |
| glrlm | HighGrayLevelRunEmphasis | 0.896 |
| firstorder | Minimum | 0.895 |
| glcm | Autocorrelation | 0.894 |
| gldm | HighGrayLevelEmphasis | 0.893 |
| glszm | LargeAreaLowGrayLevelEmphasis | 0.872 |
| glrlm | LongRunHighGrayLevelEmphasis | 0.859 |
| gldm | LargeDependenceHighGrayLevelEmphasis | 0.840 |
| shape | Flatness | 0.803 |
| glszm | LowGrayLevelZoneEmphasis | 0.795 |
| ngtdm | Coarseness | 0.786 |
| gldm | SmallDependenceLowGrayLevelEmphasis | 0.772 |
| glcm | Idmn | 0.772 |
| glszm | ZoneVariance | 0.752 |
| glszm | LargeAreaEmphasis | 0.752 |
| **glszm** | **SmallAreaLowGrayLevelEmphasis** | **0.737** |
| **shape** | **Elongation** | **0.674** |
| **glrlm** | **LongRunLowGrayLevelEmphasis** | **0.671** |
| **gldm** | **LargeDependenceLowGrayLevelEmphasis** | **0.663** |
| **glrlm** | **ShortRunLowGrayLevelEmphasis** | **0.642** |
| **glrlm** | **LowGrayLevelRunEmphasis** | **0.631** |
| **gldm** | **LowGrayLevelEmphasis** | **0.614** |
| **ngtdm** | **Busyness** | **0.037** |

**Supplementary Table 2.** Intraclass correlation coefficient for each radiomics feature

All features, except of eight revealed an ICC ≥ 0.75 (excellent). The eight non-excellently robust features are marked in bold and were excluded for further analysis.

# **S6. Pearson correlation analysis**

**Supplementary Figure 6.** Correlation heatmap of robust radiomic features

Pearson correlation heatmap of robust (ICC3 ≥ 0.75) radiomic features is shown. Statistical analysis and data visualization was done in Python 3.7.6. using seaborn version 0.11.2. ^2^

Highly correlated features (Pearson correlation ≥ 0.95, n = 52):

Maximum2DDiameterColumn; Maximum2DDiameterRow; Maximum2DDiameterSlice; Maximum3DDiameter; MinorAxisLength; VoxelVolume; InterquartileRange; MeanAbsoluteDeviation; Mean; Median; Range; RobustMeanAbsoluteDeviation; RootMeanSquared; TotalEnergy; ClusterShade; ClusterTendency; DifferenceAverage; DifferenceEntropy; DifferenceVariance; Id; Idm; JointAverage; JointEnergy; JointEntropy; MCC; MaximumProbability; SumAverage; SumEntropy; SumSquares; DependenceNonUniformity; GrayLevelVariance [gldm]; HighGrayLevelEmphasis; LargeDependenceEmphasis; SmallDependenceEmphasis; SmallDependenceHighGrayLevelEmphasis; GrayLevelNonUniformity.1 [glrlm]; GrayLevelNonUniformityNormalized [glrlm]; GrayLevelVariance.1 [glrlm]; HighGrayLevelRunEmphasis; LongRunEmphasis; RunLengthNonUniformity; RunLengthNonUniformityNormalized; RunPercentage; RunVariance; ShortRunEmphasis; ShortRunHighGrayLevelEmphasis; LargeAreaHighGrayLevelEmphasis; SizeZoneNonUniformity; SmallAreaEmphasis; SmallAreaHighGrayLevelEmphasis; ZonePercentage; ZoneVariance

were dropped to reduce feature redundancy.

Consequently, the final feature set consisted of the following 47 features:

Flatness; LeastAxisLength; MajorAxisLength; MeshVolume; Sphericity; SurfaceArea; SurfaceVolumeRatio; 10Percentile; 90Percentile; Energy; Entropy; Kurtosis; Maximum; Minimum; Skewness; Uniformity; Variance; Autocorrelation; ClusterProminence; Contrast; Correlation; Idmn; Idn; Imc1; Imc2; InverseVariance; DependenceEntropy; DependenceNonUniformityNormalized; DependenceVariance; GrayLevelNonUniformity; LargeDependenceHighGrayLevelEmphasis; SmallDependenceLowGrayLevelEmphasis; LongRunHighGrayLevelEmphasis; RunEntropy; GrayLevelNonUniformity.2 [gldm]; GrayLevelNonUniformityNormalized.1 [glrlm]; GrayLevelVariance.2 [gldm]; HighGrayLevelZoneEmphasis; LargeAreaEmphasis; LargeAreaLowGrayLevelEmphasis; LowGrayLevelZoneEmphasis; SizeZoneNonUniformityNormalized; ZoneEntropy; Coarseness; Complexity; Contrast.1[ngtdm]; Strength.

**S7. Clinical features**

We assessed and calculated different clinical scores for TACE response or risk prediction based on laboratory results and radiologic reporting ^3^:

(I) Albumin-Bilirubin (ALBI) grade to assess the liver function in HCC ^4^,

(II) hepatoma arterial embolization prognostic (HAP) score for outcome prediction in TACE HCC patients ^5^,

(III) modified HAP II (mHAP-II) score ^6^,

(IV) Selection for TrAnsarterial chemoembolization TrEatment (STATE) score ^7^ and

(V) “six-and-twelve” (6&12) score for TACE recommendation in HCC patients ^8^.

| **Clinical scores** | **All** | **Train** | **Test** |
| --- | --- | --- | --- |
| ALBI-Score |  |  |  |
| 1 | 28 (45.9) | 21 (50.0) | 7 (36.8) |
| 2 | 30 (49.2) | 19 (45.2) | 11 (57.9) |
| 3 | 2 (3.3) | 2 (4.8) | 0 (0.0) |
| N/A | 1 (1.6) | 0 (0.0) | 1 (5.3) |
| HAP-Score Group |  |  |  |
| A | 30 (49.2) | 21 (50.0) | 9 (47.4) |
| B | 20 (32.8) | 14 (33.3) | 6 (31.6) |
| C | 9 (14.8) | 6 (14.3) | 3 (15.8) |
| D | 1 (1.6) | 1 (2.4) | 0 (0.0) |
| N/A | 1 (1.6) | 0 (0.0) | 1 (5.3) |
| mHAP-II-Score Group |  |  |  |
| A | 11 (18.0) | 7 (16.7) | 4 (21.1) |
| B | 30 (49.2) | 21 (50.0) | 9 (47.4) |
| C | 11 (18.0) | 9 (21.4) | 2 (10.5) |
| D | 8 (13.1) | 5 (11.9) | 3 (15.8) |
| N/A | 1 (1.6) | 0 (0.0) | 1 (5.3) |
| State-Score-Group |  |  |  |
| low risk | 7 (11.5) | 6 (14.3) | 1 (5.3) |
| high risk | 53 (86.9) | 36 (85.7) | 17 (89.5) |
| N/A | 1 (1.6) | 0 (0.0) | 1 (5.3) |
| 6 and 12 Group |  |  |  |
| 1 | 24 (39.3) | 18 (42.9) | 6 (31.6) |
| 2 | 25 (41.0) | 16 (38.1) | 9 (47.4) |
| 3 | 12 (19.7) | 8 (19.0) | 4 (21.1) |

**Supplementary Table 3.** Clinical risk scores

Clinical risk scores are shown for the complete cohort (all), the training and the testing set.

Further, we assessed the hypervascularization of HCC-lesions by three independent raters (OE, SB, SM). The visual assessment of arterial hypervascularization was scored on a Likert scale ranging from 1 (minimal) to 5 (strong) and yielded an inter-reader ICC3 of 0.86 as calculated using the Pingouin package in Python ^9^. The visual scores of rater 1 were applied in the machine learning algorithms. The clinical scores and the visual ratings were used as clinical benchmark.

# **S8. Feature selection and model development**

**Dataset preparation**: The whole dataset was split into training and validation/ testing sets on the patient level to ensure that all lesions of a patient were distributed in the same set. The split was done at random with 70% of patients drawn for training and 30% for testing/ validation with similar proportion of responders and non-responders in the training and test set. This random split was locked and used for all analyses.

**Feature selection pool and workflow**: We used a total of seven different feature selection strategies composed of three different selection methods: (I) least absolute shrinkage and selection operator (LASSO), (II) recursive feature elimination (RFE) and (III) recursive feature addition (RFA). The RFE and RFA were used as implemented in shap-hypetune (<https://github.com/cerlymarco/shap-hypetune>; accessed: 12.04.2022) with the gradient boosting classifier LGBMClassifier ^10^ with three different boosting types: (I) “gbdt” = gradient boosting decision tree, (II) “dart” = dropouts meet multiple additive regression trees, (III) “goss” = gradient-based one-side sampling leading to three different RFE and three different RFA selection methods. LASSO was optimized using GridSearchCV with five-fold cross-validation to tune alpha according to the negative mean squared error. Non-zero features were selected. RFE and RFA was used within shap-hypetune (https://github.com/cerlymarco/shap-hypetune; accessed: 12.04.2022) for simultaneous hyperparameter tuning and feature selection with Boost-RFE and Boost-RFA to automatically select the optimal number of features. The respective single feature set with the highest accuracy was used from the RFE model, the RFA model and the non-zero LASSO features to obtain a total of three different feature subsets for each of our feature groups: radiomics, clinical and combined. If multiple selection methods yielded an equivalent accuracy, the feature subset with the smallest number of features was taken. The feature selection of the combined model was equivalent to the radiomics model. Therefore, we manually defined the combined feature set by using the feature subsets of the best working radiomics model and the best working clinical score model. The selected feature subsets are depicted in Table 2 in the manuscript.

**Classifier optimization and model selection**: Each selected feature subset was used to train and optimize a total of 7 different classifiers: (1) random forest, (2) support vector classifier, (3) extra-trees classifier, (4) AdaBoost classifier, (5) gradient boosting classifier, (6) stochastic gradient descent classifier and (7) xgboost classifier. We used Hyperopt ^11^ to perform 50 random selections of the 7 classifiers with 50 points of evaluation using Tree of Parzen Estimators to stratify the best working model. We compared the performance of each model of each selected feature subset to identify and select the feature set and optimized classifier with best performance to predict individual target lesion response. Table 3 in the manuscript summarizes the results for each classifier.

The best working model was:

RandomForestClassifier(bootstrap=False, criterion='entropy', max_features=None, min_samples_leaf=2, n_estimators=165, n_jobs=1, random_state=3, verbose=False)

The model was locked and used to predict the response to TACE on the patient level. The selected feature subset was used to train and test a random survival forest using Scikit-survival 0.16.1 ^12^ to test the performance of the features to predict the overall survival of the patients.

# **S9. Overall survival**


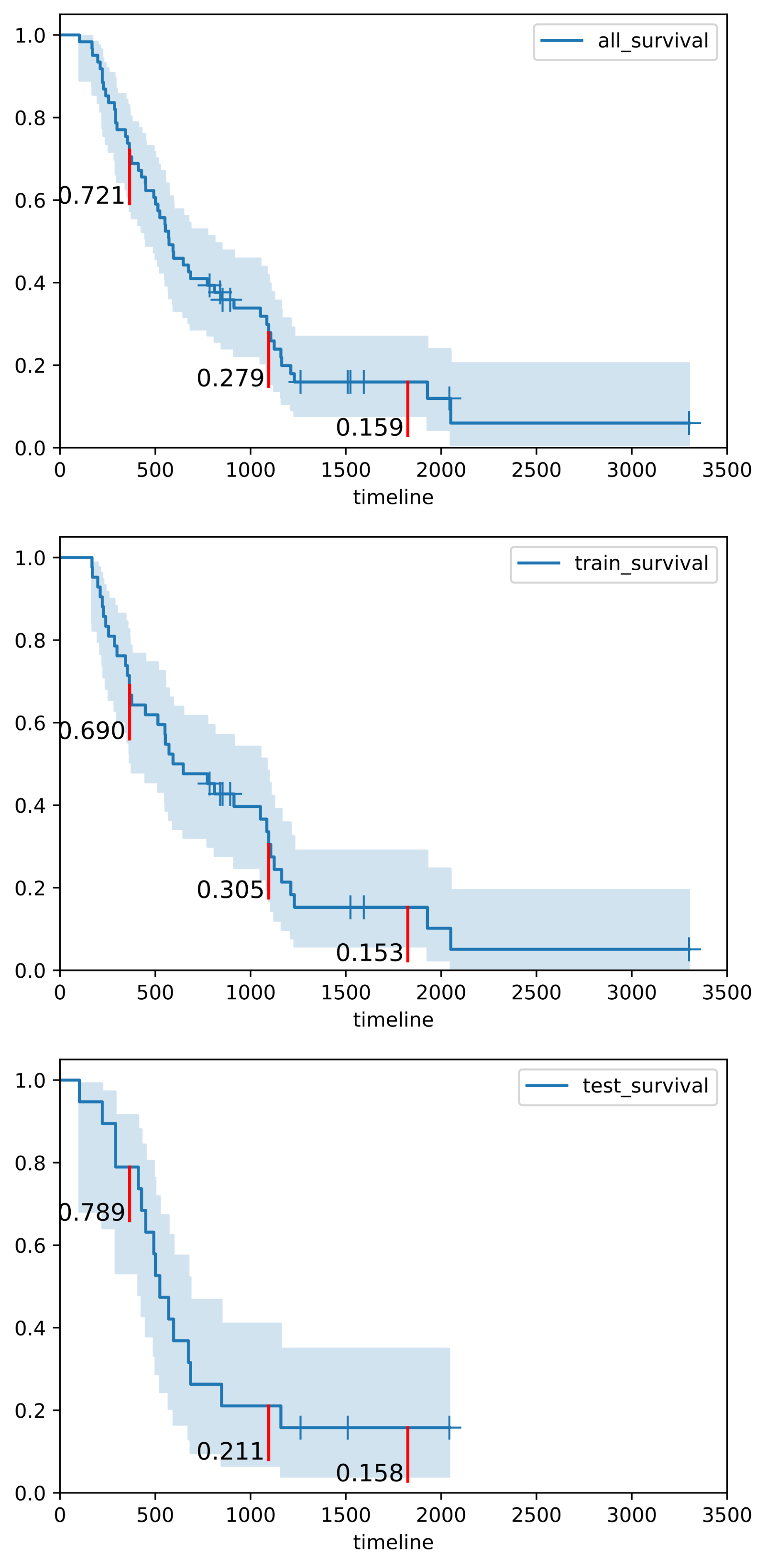


**Supplementary Figure 7.** Kaplan-Meier survival plots

Red lines depict the proportion of estimated living patients at the timepoints: 1 year, 3 years and 5 years.

# **S10. Low dimensional embedding did not identify response clusters**


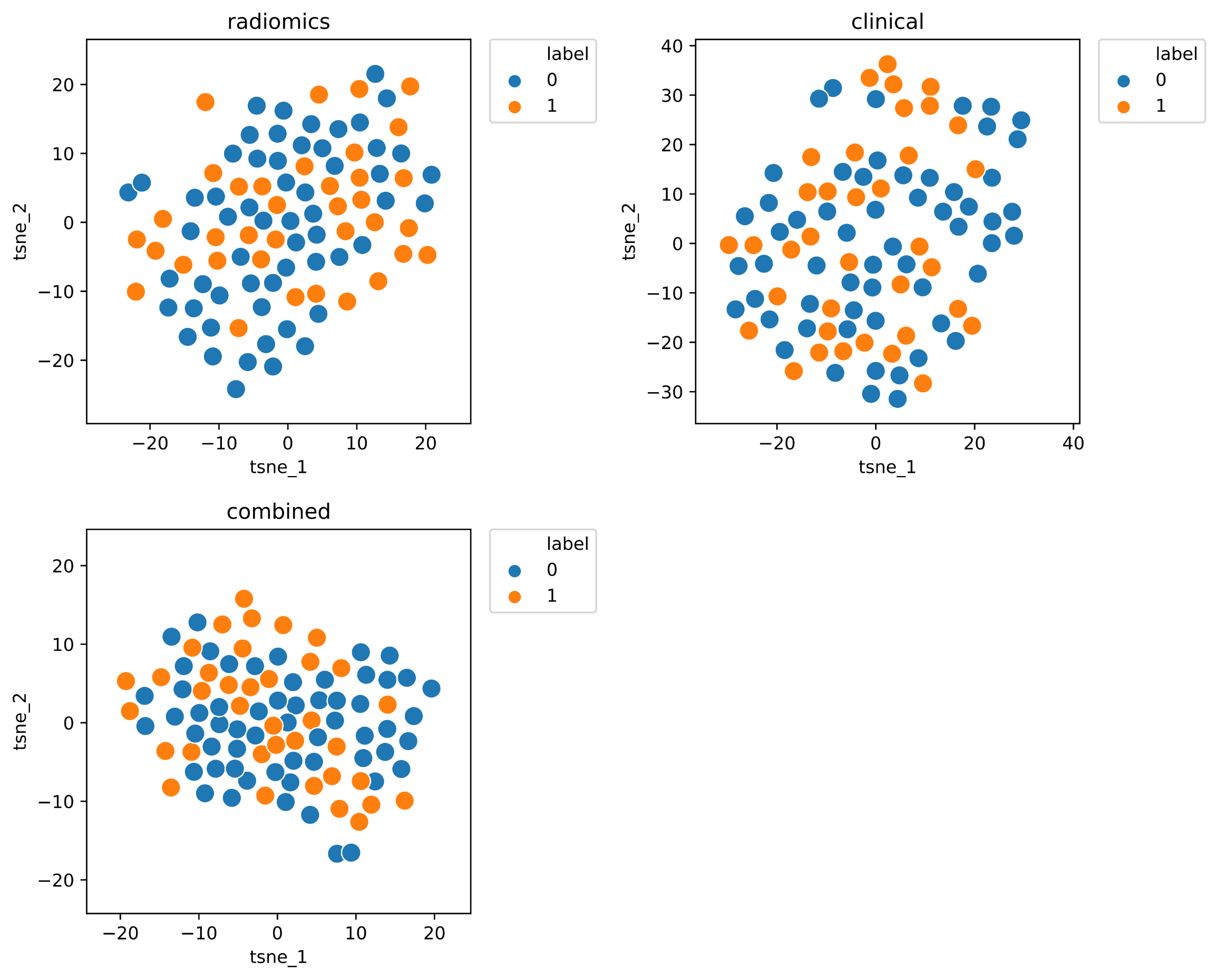


**Supplementary Figure 8.** T-distributed stochastic neighbor embedding (t-SNE) plots

T-distributed stochastic neighbor embedding (t-SNE) using the radiomics features, clinical features or the combined features set. Target-labels were defined as response (complete or partial mRECIST response, label = 1, orange) versus no response (stable or progressive mRECIST disease, label = 0, blue). The lesion-wise embedding is shown and now clustering is revealed.

# **References**

1. Lambin, P. *et al.* Radiomics: the bridge between medical imaging and personalized medicine. *Nat. Rev. Clin. Oncol.* **14**, 749–762 (2017).

2. Waskom, M. Seaborn: Statistical Data Visualization. *J. Open Source Softw.* **6**, 3021 (2021).

3. Müller, L. *et al.* Current Strategies to Identify Patients That Will Benefit from TACE Treatment and Future Directions a Practical Step-by-Step Guide. *J. Hepatocell. Carcinoma* **8**, 403–419 (2021).

4. Johnson, P. J. *et al.* Assessment of liver function in patients with hepatocellular carcinoma: A new evidence-based approach - The ALBI grade. *J. Clin. Oncol.* **33**, 550–558 (2015).

5. Kadalayil, L. *et al.* A simple prognostic scoring system for patients receiving transarterial embolisation for hepatocellular cancer. *Ann. Oncol.* **24**, 2565–2570 (2013).

6. Park, Y. *et al.* Addition of tumor multiplicity improves the prognostic performance of the hepatoma arterial-embolization prognostic score. *Liver Int.* **36**, 100–107 (2016).

7. Hucke, F. *et al.* How to STATE suitability and START transarterial chemoembolization in patients with intermediate stage hepatocellular carcinoma. *J. Hepatol.* **61**, 1287–1296 (2014).

8. Wang, Q. *et al.* Development of a prognostic score for recommended TACE candidates with hepatocellular carcinoma: A multicentre observational study. *J. Hepatol.* **70**, 893–903 (2019).

9. Vallat, R. Pingouin: statistics in Python. *J. Open Source Softw.* **3**, 1026 (2018).

10. Ke, G. *et al.* LightGBM: A highly efficient gradient boosting decision tree. *Adv. Neural Inf. Process. Syst.*, 3147–3155 (2017).

11. Bergstra, J., Yamis, D. & Cox, D. D. Making a Science of Model Search: Hyperparameter Optimization in Hundreds of Dimensions for Vision Architectures. *TProc. 30th Int. Conf. Mach. Learn. (ICML 2013)* I-115 to I–23

12. *S. Pölsterl, “scikit-survival: A Library for Time-to-Event Analysis Built on Top of scikit-learn,” Journal of Machine Learning Research, vol. 21, no. 212, pp. 1–6, 2020*.
